# Dynamic coronary roadmap-guided versus traditional percutaneous coronary intervention techniques in contrast medium volume reduction: a systematic review and meta-analysis

**DOI:** 10.1186/s43044-026-00763-2

**Published:** 2026-07-01

**Authors:** Amjad Almansi, Shahd Alqato, Lama Hossam Taher, Abdelrahman M. Elettreby, Suhel F. Batarseh, Nada Mostafa Al-dardery, Zina Otmani, Salem Elshenawy, Mohamed Abouzid

**Affiliations:** 1 Prince Hamza Hospital, Amman, Jordan; 2 Arab Medical Center, Amman, Jordan; 3 Ain Shams University, Cairo, Egypt; 4 Mansoura University, Al Mansurah, Egypt; 5 Jordan University of Science and Technology, Irbid, Jordan; 6 Fayoum University, Al Fayyum, Egypt; 7 Mouloud Mammeri University of Tizi-Ouzou, Tizi Ouzou, Algeria; 8 Alexandria University, Alexandria, Egypt; 9 Poznan University of Medical Sciences, Poznań, Poland

**Keywords:** Angiography, CAD, PCI, Contrast media, AKI

## Abstract

**Background:**

Dynamic Coronary Roadmap (DCR) is a novel software providing a motion-compensated, real-time overlay of coronary arteries to assist PCI device navigation with a single contrast injection. This meta-analysis evaluated the effectiveness and safety of DCR.

**Methods:**

We searched PubMed, Scopus, Web of Science, Cochrane Library, and Embase from inception to May 5, 2024. Outcomes were pooled as risk ratios (RR) or mean differences (MD) with 95% confidence intervals (CI) using random-effects models. PROSPERO registration: CRD42024553689.

**Results:**

Eight studies (two RCTs, six observational; 1,512 patients) were included. DCR-guided PCI significantly reduced contrast media volume (MD =  − 45.82 mL, 95% CI − 68.33 to − 23.30; *P* < 0.001; I^2^ = 97%), air kerma (MD =  − 283.09 mGy, 95% CI − 474.47 to − 91.70; *P* = 0.004; I^2^ = 99%), dose area product (MD =  − 6.85 Gy/cm^2^, 95% CI − 10.48 to − 3.22; *P* = 0.0002; I^2^ = 82%), radiation duration (MD =  − 2.79 min, 95% CI − 4.59 to − 0.98; *P* = 0.002; I^2^ = 94%), and procedural duration (MD =  − 3.51 min, 95% CI − 6.69 to − 0.32; *P* = 0.03; I^2^ = 0%). No significant differences were found in procedural success (RR = 1.00; 95% CI 0.99–1.01; I^2^ = 0%) or acute kidney injury incidence (RR = 0.40; 95% CI 0.15–1.08; I^2^ = 0%). Risk of bias was mixed for RCTs (low to high risk across domains) and moderate to high for observational studies, with most scoring ≥ 7 on the Newcastle–Ottawa Scale.

**Conclusion:**

DCR-guided PCI was associated with reductions in contrast use and radiation metrics without compromising procedural success; however, the overall certainty of evidence is low due to study design limitations, risk of bias, and substantial heterogeneity, warranting cautious interpretation.

**Graphical abstract:**

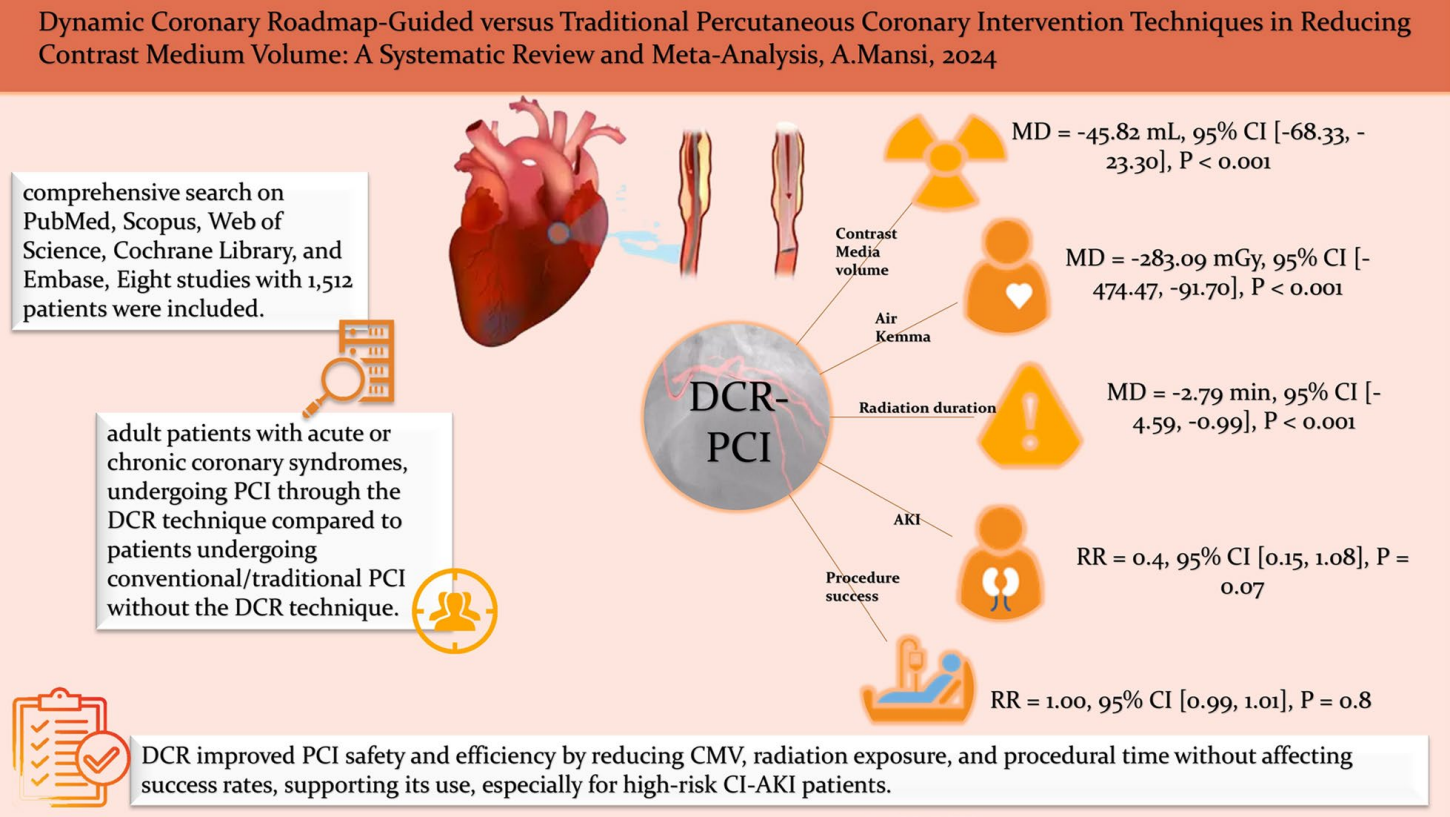

**Supplementary Information:**

The online version contains supplementary material available at 10.1186/s43044-026-00763-2.

## Introduction

Percutaneous coronary intervention (PCI) is a cornerstone revascularization technique for patients with obstructive coronary artery disease. By mechanically dilating or stenting stenotic coronary segments, PCI improves myocardial perfusion and relieves ischemia, with generally favorable procedural outcomes and a low complication rate when performed in appropriate candidates [1, 2]. Despite these advantages, PCI relies on intravascular contrast media (CM) to delineate coronary anatomy, assess lesion severity, and guide device delivery. While indispensable for visualization, iodinated CM carries potential nephrotoxic and ischemic risks, particularly affecting renal tubular cells, and is a well-recognized precipitant of contrast-induced acute kidney injury (CI-AKI) [3].

CI-AKI remains one of the most important non-cardiac complications of PCI, with reported incidence ranging from approximately 2% in low-risk patients to over 20% in those with chronic kidney disease, diabetes, or undergoing complex interventions [4]. Its occurrence is associated with longer hospital stays, higher healthcare costs, and significantly worse outcomes, including increased in-hospital and long-term mortality, major adverse cardiac events (MACE), and major adverse cardiac and cerebrovascular events (MACCE)—even when the index PCI is technically successful [5].

Prevention of CI-AKI is therefore a procedural priority. Although strategies such as periprocedural hydration, the use of low- or iso-osmolar CM, and pharmacologic adjuncts (e.g., N-acetylcysteine, sodium bicarbonate) have been explored, the most consistently supported intervention is limiting CM volume [2, 3, 6, 7]. A direct, dose-dependent relationship exists between CM exposure and CI-AKI risk, with larger volumes increasing osmotic load and prolonging toxic exposure to renal tissue [6, 7].

In routine PCI, operators rely on fluoroscopy and intermittent angiographic “puffs” to maintain spatial awareness of the target vessel. Because opacification from a single injection is short-lived, repeated CM injections are often necessary, especially in complex or prolonged cases [8]. To address this limitation, Philips Healthcare (Best, Netherlands) has developed the Dynamic Coronary Roadmap (DCR), a commercially available navigation support system. DCR generates a dynamic, motion-compensated overlay of a previously acquired coronary angiogram on live fluoroscopy, providing continuous visual feedback throughout device manipulation [9, 10]. By reducing the need for repeated contrast injections, DCR has the potential to meaningfully lower CM usage while maintaining procedural precision in device positioning and stent deployment.

Early clinical studies have reported favorable procedural metrics with DCR. Still, its overall impact on contrast reduction, radiation exposure, procedural efficiency, and safety outcomes across diverse patient populations has not been systematically synthesized. Accordingly, we conducted a systematic review and meta-analysis to compare DCR-guided PCI with conventional angiography-guided PCI, focusing on effectiveness and safety.

## Methods

### Protocol registration

This systematic review and meta-analysis was conducted in accordance with the Preferred Reporting Items for Systematic Reviews and Meta-Analyses (PRISMA) statement [11] and methodological recommendations outlined in the Cochrane Handbook for Systematic Reviews of Interventions [12]. The study protocol was prospectively registered in the International Prospective Register of Systematic Reviews (PROSPERO) under registration number CRD42024553689.

### Data sources & search strategy

A comprehensive search of PubMed, Scopus, Web of Science, Cochrane Library, and Embase was performed from database inception to May 5, 2024, with no language or date restrictions applied at the search stage. The search strategy combined controlled vocabulary and free-text terms related to dynamic coronary roadmap and percutaneous coronary intervention, using Boolean operators. The core search terms included:

(((roadmap AND fusion AND imaging) OR (dynamic AND coronary AND roadmap) OR (DCR)) AND ((angiography) OR (arteriography) OR (angiogram) OR (percutaneous AND coronary AND intervention) OR (percutaneous AND coronary AND revascularization) OR (PCI) OR (coronary AND angiography))).

In addition, we conducted manual searches using Google and Google Scholar with the terms “Dynamic coronary roadmap,” “percutaneous coronary intervention,” and “contrast volume.” Reference lists of all included studies and relevant reviews were also screened to identify additional eligible publications. The full electronic search strategy is provided in Supplementary Table 1.

### Eligibility criteria

Studies were selected according to a pre-defined PICOS framework:

- Population (P): Adults with acute or chronic coronary syndromes undergoing PCI.
- Intervention (I): PCI performed with DCR guidance.
- Comparator (C): Conventional/traditional PCI without DCR assistance.
- Outcomes (O): The primary outcome was contrast media volume (CMV). Secondary outcomes included radiation exposure parameters (air kerma and dose–area product), radiation duration, procedural duration, procedural success, and AKI.
- Study Design (S): Randomized controlled trials (RCTs) or observational cohort studies (prospective or retrospective) reporting at least one outcome of interest.

We excluded animal studies, non-English publications, single-arm designs, case reports, conference abstracts, protocols, book chapters, and narrative commentaries.

### Study selection and data extraction

Two independent reviewers screened all titles and abstracts for potential eligibility, followed by a full-text review of shortlisted articles. Disagreements were resolved through discussion or consultation with a third reviewer. Data were extracted using a standardized form capturing:

1. Study-level characteristics (country, setting, design, sample size)
2. Baseline participant demographics and clinical characteristics
3. Risk-of-bias assessment results
4. Reported outcomes and statistical measures

### Risk of bias assessment

The methodological quality of RCTs was evaluated using the Cochrane Risk of Bias 2.0 (ROB 2) tool [13], which assesses bias across randomization, allocation concealment, blinding, attrition, outcome reporting, and other potential domains. Observational studies were appraised using the Newcastle–Ottawa Scale (NOS) [14], which rates quality based on selection, comparability, and outcome assessment. Both assessments were performed independently by two reviewers, with any discrepancies adjudicated by a third reviewer.

### Effect measures and statistical analysis

All statistical analyses were conducted using Review Manager (RevMan) version 5.4 [15]. For continuous outcomes, pooled effects were expressed as mean differences (MD) with 95% confidence intervals (CI). When studies reported medians and interquartile ranges (IQR), means and standard deviations (SD) were estimated using the method of Wan et al. [16]; for medians with ranges, the method of Hozo et al. [17] was applied. For dichotomous outcomes, pooled effects were expressed as risk ratios (RR) with 95% CI. Statistical heterogeneity was assessed using the I^2^ statistic. An I^2^ ≥ 50% was considered indicative of substantial heterogeneity. In such cases, a random-effects model was applied to account for between-study variability. For outcomes with low statistical heterogeneity (I^2^ < 50%), a fixed-effect model was used, under the assumption that studies were estimating a common underlying effect. The analytical strategy, including model selection based on heterogeneity thresholds, was pre-specified before data synthesis to minimize the risk of selective reporting or analytical bias. Statistical significance was set at *p* < 0.05. Sensitivity analyses were conducted by iteratively excluding each included study to evaluate the robustness of the pooled results.

## Results

### Literature search

**Fig. 1:**
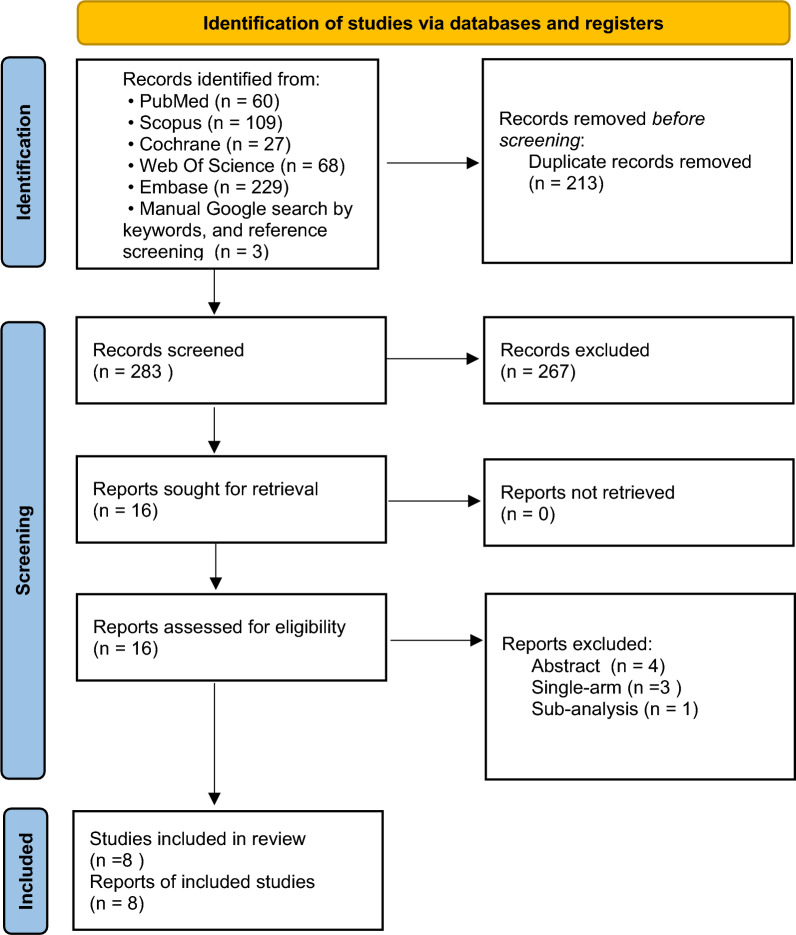
PRISMA 2020 flow diagram for new systematic reviews, which included searches of databases and registers only

The initial database search identified 496 records. After duplicate removal and title/abstract screening, 16 articles underwent full-text review. Eight studies fulfilled the eligibility criteria and were included in the meta-analysis (Fig. 1). Of these, two were RCTs, and six were observational cohort studies.

### Characteristics of included studies

The pooled analysis included 1,512 patients—723 treated with DCR-guided PCI and 787 undergoing conventional PCI. Study locations included Egypt (n = 3), Japan (n = 2), Germany (n = 1), India (n = 1), and one multicenter trial spanning Belgium, Israel, Spain, and the USA. The mean participant age was 64 years (SD 10). Detailed study-level characteristics are provided in Table 1, and patient baseline characteristics in Table 2.

**Table 1:** Study summary of the included studies

Study	Design	Country	Centers (n)	Sample size (n)	Inclusion criteria	Exclusion criteria	Primary outcomes	Key findings
Bendary et al. [31]	Prospective cohort	Egypt	1	80	Patients ≥ 18 years with chronic coronary syndrome refractory to medical therapy	Hemodialysis, ACS, CTO lesions	CMV, fluoroscopy time, AK, DAP, angiographic and procedural success, post-PCI eGFR	DCR significantly reduced CMV, fluoroscopy time, AK, and DAP (all *p* < 0.001). Post-PCI eGFR was higher in the DCR group (*p* = 0.015). No differences in procedural characteristics (e.g., IVUS use, DES implantation)
Hennessey et al. [23]	RCT	Multicenter (Belgium, Israel, Spain, USA)	6	356	Adults undergoing complex PCI requiring > 25 mL contrast	Emergency PCI, STEMI, CTO, severe CKD, OCT use, atherectomy, pregnancy	Total CMV	DCR significantly reduced contrast use and number of cineangiograms (*p* < 0.001). No differences in radiation, procedural duration, AKI, procedural success, or in-hospital MACE
Piayda et al. [29]	RCT	Germany	1	133	Patients with type A/B coronary lesions undergoing elective PCI	Language barrier, limited life expectancy, inability to follow up	CMV	Contrast exposure was significantly lower with DCR (*p* < 0.001). No differences in procedure duration, radiation time, or DAP
Hirano et al. [34]	Retrospective cohort	Japan	1	275	Patients with CKD and stable angina	CTO, graft lesions, dialysis, emergency/ad hoc PCI	Composite clinical outcomes (death, HF, MI), dialysis initiation	DCR reduced CMV and radiation (*p* < 0.01). Lower incidence of adverse outcomes and dialysis initiation in DCR group
Humane et al. [33]	Retrospective cohort	India	1	338	All-comer patients undergoing PCI	Not reported	CMV, DAP, fluoroscopy time	DCR significantly reduced CMV (*p* = 0.02), DAP (*p* = 0.003), and fluoroscopy time (*p* = 0.0006)
Maher et al. [24]	Prospective cohort	Egypt	1	40	Not reported	Complex PCI, CTO, emergent PCI, CKD, contrast allergy	CMV, radiation dose, procedural outcomes, complications	DCR reduced CMV (*p* < 0.001), radiation dose (*p* = 0.026), and serum creatinine (*p* = 0.015). No difference in complications or procedural duration
Mansy et al. [32]	Prospective cohort	Egypt	1	193	Patients with complex lesions and expected high contrast usage	ACS, CTO with unknown anatomy, infection, pregnancy	CMV, AK, DAP, fluoroscopy time	DCR significantly reduced CMV, AK, DAP, and fluoroscopy time (all *p* < 0.001)
Yabe et al. [2]	Retrospective cohort	Japan	1	130	PCI using Azurion imaging system	CTO, ACS, hemodialysis	CMV, radiation metrics	DCR reduced CMV (*p* = 0.006) and fluoroscopy time (*p* = 0.007). No significant difference in AK or DAP; no significant change in renal function

AK, Air kerma; ACS, Acute coronary syndrome; CKD, Chronic kidney disease; CMV, Contrast media volume; CTO, Chronic total occlusion; DAP, Dose–area product; DCR, Dynamic coronary roadmap; DES, Drug-eluting stent; eGFR, Estimated glomerular filtration rate; HF, Heart failure; IVUS, Intravascular ultrasound; LMCA, Left main coronary artery; MACE, Major adverse cardiovascular events; MI, Myocardial infarction; OCT, Optical coherence tomography; PCI, Percutaneous coronary intervention; RCA, Right coronary artery; RCT, Randomized controlled trial

**Table 2:** Baseline characteristics of the studies included

Study ID	Arms	N	Age (Mean ± SD)	Females n (%)	HTN n (%)	DM n (%)	CKD n (%)	Prior MI n (%)	Prior CABG n (%)	Treated vessels n (%)
Bendary et al. [31]	DCR	40	57 ± 10	18 (45)	27 (67.5)	29 (72.5)	10 (25)	10 (25)	1 (2.5)	LAD: 22 (55) LCX: 9 (22.5) RCA: 7 (17.5) Left Main: 2 (5)
	Normal PCI	40	59 ± 10	16 (40)	27 (67.5)	26 (65)	0 (0)	16 (40)	4 (10)	LAD: 16 (40) LCX: 12 (30) RCA: 9 (22.5) Left Main: 3 (7.5)
Hennessey et al. [23]	DCR	179	65.8 ± 10.8	36 (20.1)	135 (75.4)	66 (36.9)	18 (10.1)	55 (30.7)	12 (6.7)	LAD: 91 (50.8) LCX: 52 (29.1) RCA: 49 (27.4) Left Main:5 (2.8)
	Normal PCI	177	65.9 ± 10.9	38 (21.5)	124 (70.1)	68 (38.4)	26 (14.7)	54 (30.5)	15 (8.5)	LAD: 89 (50.3) LCX: 58 (32.8) RCA: 49 (27.7) Left Main: 4 (2.3)
Piayda et al. [29]	DCR	66	mid-60's*	42 (31.5)	–	–	–	–	–	–
	Normal PCI	67			–	–	–	–	–	–
Hirano et al. [34]	DCR	113	75.54 ± 10.3	13 (11.5)	100 (88.5)	63 (55.8)	113 (100)	53 (46.9)	7 (6.2)	LAD: 59 (52.2) LCX: 26 (23) RCA: 25 (22.1) Left Main: 3 (2.7)
	Normal PCI	113	72.39 ± 9.44	21 (18.6)	91 (80.5)	57 (50.4)	113 (100)	44 (38.9)	6 (5.3)	LAD: 60 (53.1) LCX: 26 (23) RCA: 25 (22.1) Left Main: 2 (1.8)
Humane et al. [33]	DCR	169	56.8 ± 11.5	62 (36.9)	77. (45.6)	39 (23.1)	–	–	–	–
	Normal PCI	169	57.9 ± 11.8	68 (40.2)	78 (46.2)	40 (23.7)	–	–	–	–
Maher et al. [24]	DCR	20	59.35 ± 8.00	4 (20)	14 (70)	11 (55)	0	–	–	–
	Normal PCI	20	56.95 ± 8.89	9 (45)	16 (80)	12 (60)	0	–	–	–
Mansy et al. [32]	DCR	93	61.19 ± 8.25	24 (25.8)	–	–	–	–	–	LAD: 67 (72) LCX: 35 (37.6) RCA: 42 (45.2) Left Main: 6 (6.5)
	Normal PCI	100	60.55 ± 7.04	27 (27)	–	–	–	–	–	LAD: 69 (69) LCX: 38 (38) RCA: 47 (47) Left Main: 3 (3)
Yabe et al. [2]	DCR	38	74.84 ± 12.11	11 (29)	26 (68.4)	14 (36.8)	8 (21)	3 (7.8)	0	LAD:21 (48.8) LCX:15 (34.9) RCA: 6 (14.0) Left Main: 1 (2.3)
	Normal PCI	92	73.52 ± 10.85	28 (30.5)	73 (79.3)	32 (34.7)	30 (30.6)	11 (11.9)	0	LAD: 50 (48.5) LCX: 24 (23.3) RCA: 28 (27.2) Left Main: 1 (1)

Study ID	Lesion characteristics				Procedural characteristics				
	Type of lesion n (%)	CAC n (%)	Bif n (%)	ISR. n (%)	Elective or Ad hoc PCI n (%)	IVUS use n (%)	Pre-dil. n (%)	DES implantation n (%)	N of stents n (%)
Bendary et al. [31]	A: 28 (70) B: 9 (22.5) B2: 3 (7.5) C: 0	8 (20)	7 (17.5)	5 (12.5)	–	3 (7.5)	23 (57.5)	39 (97.5)	1: 30 (76.9) 2: 9 (23.1) 3: 0
	A: 26 (65) B: 10 (25) B2: 4 (10) C: 0	12 (30)	9 (22.5)	6 (15)	–	7 (17.5)	26 (65)	40 (100)	1: 31 (77.5) 2: 8 (20) 3: 1 (2.5)
Hennessey et al. [23]	–	30 (16.8)	41 (22.9)		Ad hoc: 110 (61.5) Elective: 69 (38.5)	73 (40.8)	–	–	0: 4 (2.2) 1: 121 (67.6) 2: 40 (22.3) 3: 9 (5.0) 4: 3 (1.7) 5: 2 (1.1)
	–	26 (14.7)	53 (29.9)		Ad hoc: 116 (65.5) Elective: 61 (34.5)	60 (33.9)	–	–	0: 1 (0.6) 1: 111 (62.7) 2: 44 (24.9) 3: 18 (10.2) 4: 2 (1.1) 5: 1 (0.6)
Piayda et al. [29]	Type A or type B: 133	–	–	–	Ad hoc: 0,0 Elective: 133 (100)	–	66 (100)	66 (100)	1: 62 (93.7)
		–	–	–		–	67 (100)	67 (100)	1: 59 (88.1)
Hirano et al. [34]	A: 3 (2.7) B1: 12 (10.6) B2: 46 (40.7) C: 52 (46)	52 (46.0)	38 (33.6)	–	Ad hoc: 0 Elective: 113 (100)	103 (91.2)	9 (8)	103 (91.2)	–
	A: 3 (2.7) B1: 19 (16.8) B2: 56 (49.6) C: 47 (41.6)	47 (41.6)	49 (43.4)	–	Ad hoc: 0 Elective: 113 (100)	104 (92)	8 (7.1)	102 (90.3)	–
Humane et al. [33]	–	–	–	–	Ad hoc: 25 (14.7) Elective: 144 (85.2)	–	–	–	1: 106 (62.7) 2: 43 (25.4) = / > 3: 20 (11.8)
	–	–	–	–	Ad hoc: 28 (16.6) Elective: 141 (83.4)	–	–	–	1: 104 (61.5) 2: 51 (30.2) = / > 3: 14 (8.3)
Maher et al. [24]	–	–	–	–	–	–	–	–	1.45 ± 0.75**
	–	–	–	–	–	–	–	–	1.6 ± 0.68**
Mansy et al. [32]	A: 8 (8.6) B: 84 (90.3) C: 7 (7.5)	29 (31.2)	7 (7.5)	4 (4.3)	Ad hoc: 0 Elective: 93 (100)	4 (4.3)	1.98 (0.74)	–	1.80 ± 0.75**
	A: 16 (16) B: 79 (79) C: 4 (4)	18 (18)	6 (6)	4 (4)	Ad hoc: 0 Elective: 100 (100)	4 (4)	2.07 (0.74)	–	1.66 ± 0.67**
Yabe et al. [2]	A: 12 (27.9) B1:22 (51.2) B2: 8 (18.6) C: 1 (2.3)	4 (9.3)	16 (37.2)	1 (2.3)	Ad hoc: 4 (9.3) Elective: 39 (90.7)	38 (88.3)	41 (95.3)	43 (100)	1: 38 (100)
	A: 28 (27.2) B1: 52 (50.5) B2: 20 (19.4) C: 3 (2.9)	15 (14.5)	21 (20.3)	6 (5.8)	Ad hoc: 13 (12.6) Elective: 90 (87.4)	83 (80.5)	95 (92.2)	99 (96.1)	1: 92 (100)

BMI, Body mass index; HTN, Hypertension; DM, Diabetes mellitus; CKD, Chronic kidney disease; eGFR, Estimated glomerular filtration rate; MI, Myocardial infarction; CABG, Coronary artery bypass graft; CAC, Coronary artery calcification; Bif., Bifurcation; ISR, In-stent restenosis; IVUS, Intravascular ultrasound; Pre-dil., Pre-dilation; DES, Drug-eluting stent

^*^as reported in the trial results

^**^reported as mean ± SD

### Risk of bias assessment

**Fig. 2:**
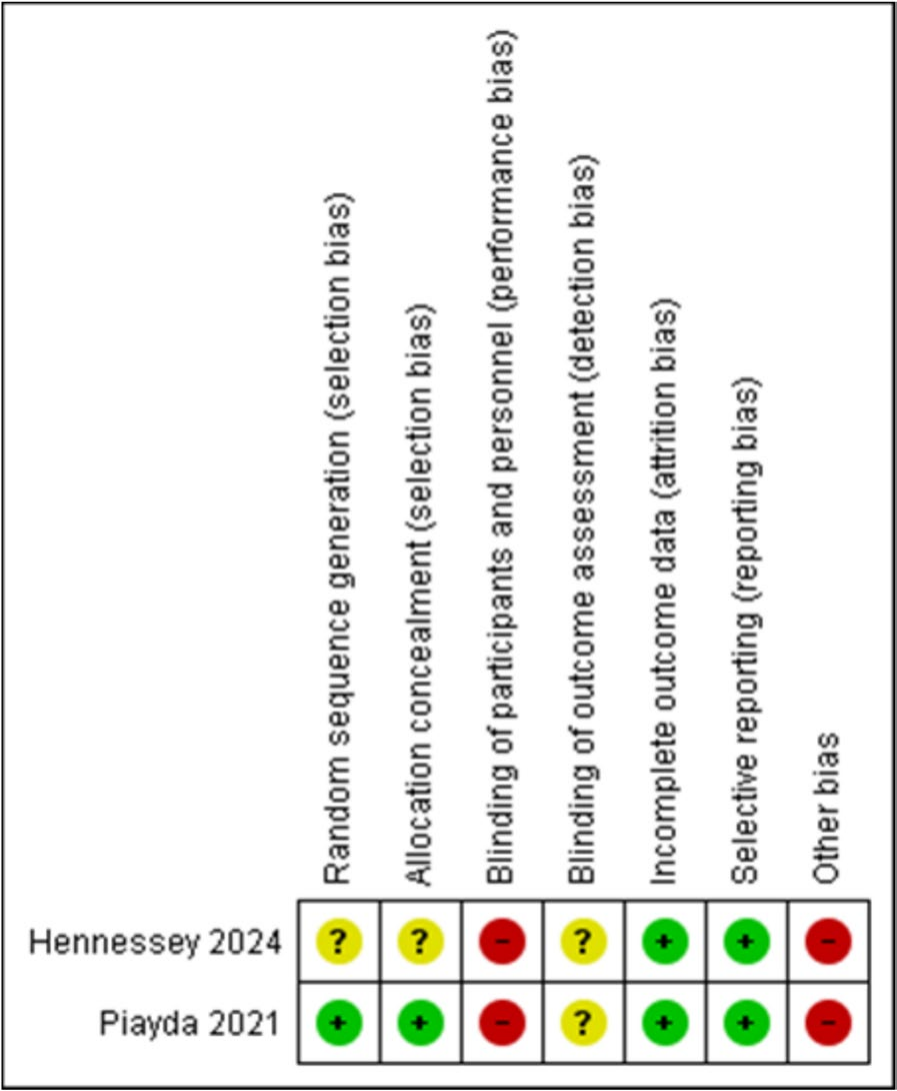
Risk of bias assessment for the randomized control trials included

Across the RCTs, the risk of bias assessment (Fig. 2) showed mixed methodological quality. Hennessey 2024 had unclear risk for random sequence generation and blinding of outcome assessment, with high risk for allocation concealment, blinding of participants/personnel, and other bias, but low risk for attrition and reporting bias. Piayda 2021 demonstrated generally lower risk, with low risk in most domains, except for unclear risk in blinding of outcome assessment and high risk for other biases.

For the observational studies (Table 3), Newcastle–Ottawa Scale ratings indicated overall moderate-to-high quality. Four studies [2, 31, 33, 34] and Maher et al. [24] scored between 7 and 9, reflecting strong selection, comparability, and outcome assessment, and were thus rated as high quality. Mansy 2024 scored 6, indicating moderate quality, mainly due to weaker representativeness and follow-up adequacy. Overall, most observational evidence was robust, though a few studies had methodological limitations that may affect interpretability.

**Table 3:** Summary of risk of bias in observational studies using the Newcastle–Ottawa Scale

Study ID	Selection				Comparability	Outcome			Overall score	Quality
	Representativeness of the exposed cohort	Selection of the non-exposed cohort	Ascertainment of exposure	The outcome of interest was not present at start of study	Comparability of cohorts	Assessment of outcome	Was follow-up long enough?	Adequacy of follow up		
Bendary et al. [31]	✵	✵	✵	✵	✵	–	✵	✵	7	High
Hirano et al. [34]	✵	✵	✵	✵	✵	✵	✵	–	7	High
Humane et al. [33]	✵	✵	✵	✵	✵✵	✵	✵	✵	9	High
Maher et al. [24]	✵	✵	✵	✵	✵✵	–	✵	✵	8	High
Mansy et al. [32]	–	✵	✵	✵	✵	–	✵	✵	6	Moderate
Yabe et al. [2]	✵	✵	✵	✵	✵✵	✵	✵	✵	9	High

✵Indicates that this domain was of low risk of bias. A study with a score ranging from 0 to 3 indicates a high risk of bias, a score of 4–6 indicates moderate risk of bias, and a score of 7–9 indicates a low risk of bias

### Primary outcome—contrast media volume (CMV)

**Fig. 3:**
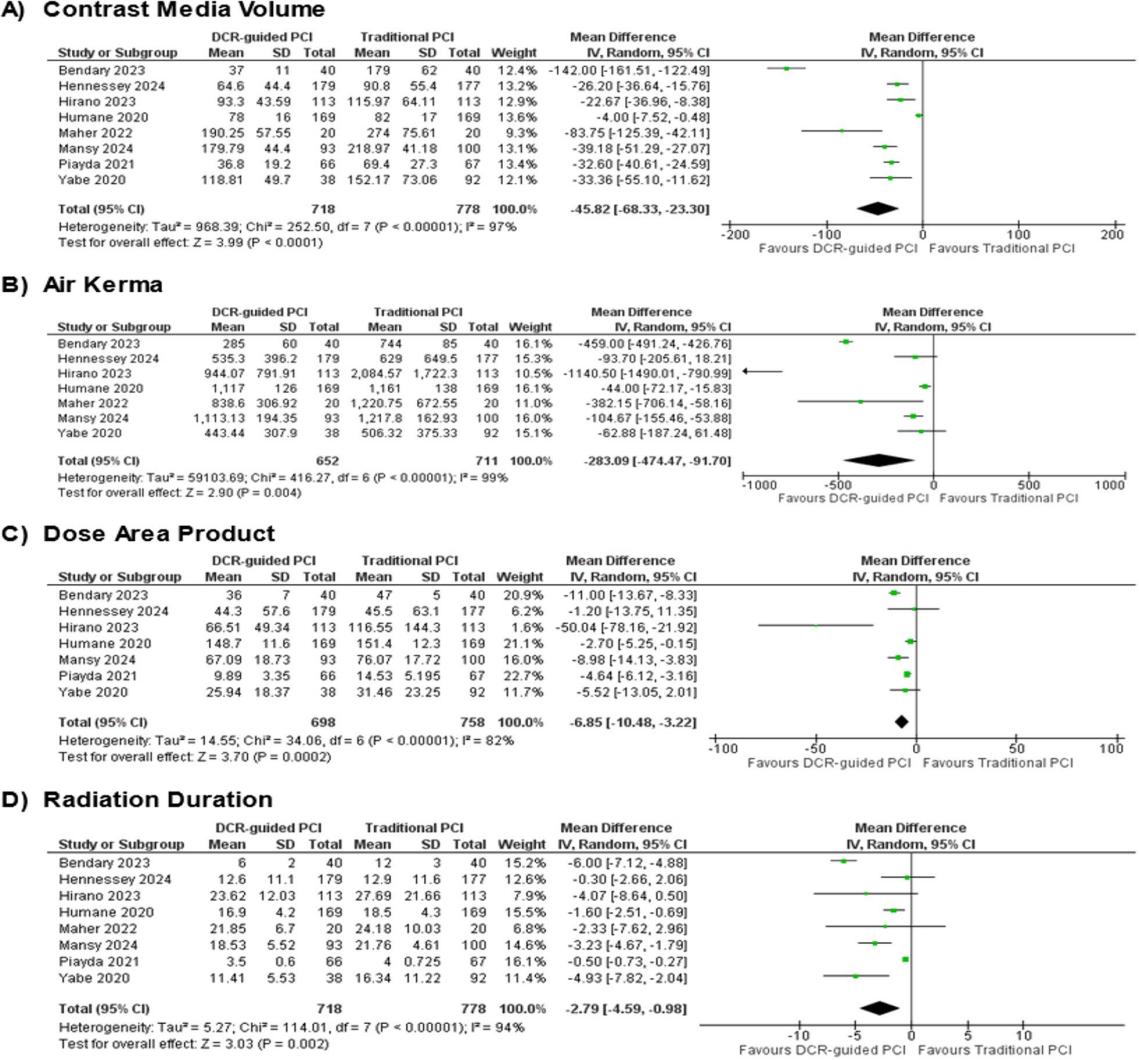
Meta-Analysis results

All eight studies (n = 1,496 patients) reported CMV. DCR-guided PCI was associated with a significantly lower CMV compared with conventional PCI (MD =  − 45.82 mL, 95% CI [− 68.33, − 23.30], *p* < 0.0001). Heterogeneity was substantial (I^2^ = 97%, *p* < 0.00001) and was not reduced by sensitivity analysis (Fig. 3A). Given the high heterogeneity, this pooled estimate should be interpreted as an average effect across diverse clinical settings rather than a precise estimate of effect size.

### Secondary outcomes

#### Radiation energy

- **Air kerma:** Seven studies (n = 1,363) demonstrated lower air kerma with DCR-guided PCI (MD =  − 283.09 mGy, 95% CI [− 474.47, − 91.70], *p* = 0.004), with considerable heterogeneity (I^2^ = 99%, *p* < 0.00001; Fig. 3B). This finding reflects a consistent direction of benefit, although the magnitude of reduction varied substantially across studies.
- **Dose–area product (DAP):** Seven studies (n = 1,456) also favored DCR-guided PCI (MD =  − 6.85 Gy/cm^2^, 95% CI [− 10.48, − 3.22], *p* = 0.0002), with high heterogeneity (I^2^ = 82%, *p* < 0.00001; Fig. 3C). Interpretation should consider variability across study protocols and patient populations.

#### Radiation duration

Eight studies (n = 1,496) demonstrated reduced radiation duration with DCR-guided PCI (MD =  − 2.79 min, 95% CI [− 4.59, − 0.98], *p* = 0.002), with substantial heterogeneity (I^2^ = 94%, *p* < 0.00001; Fig. 3D). The pooled estimate represents an overall trend favoring DCR, with variability in effect size across studies.

#### Procedural duration

**Fig. 4:**
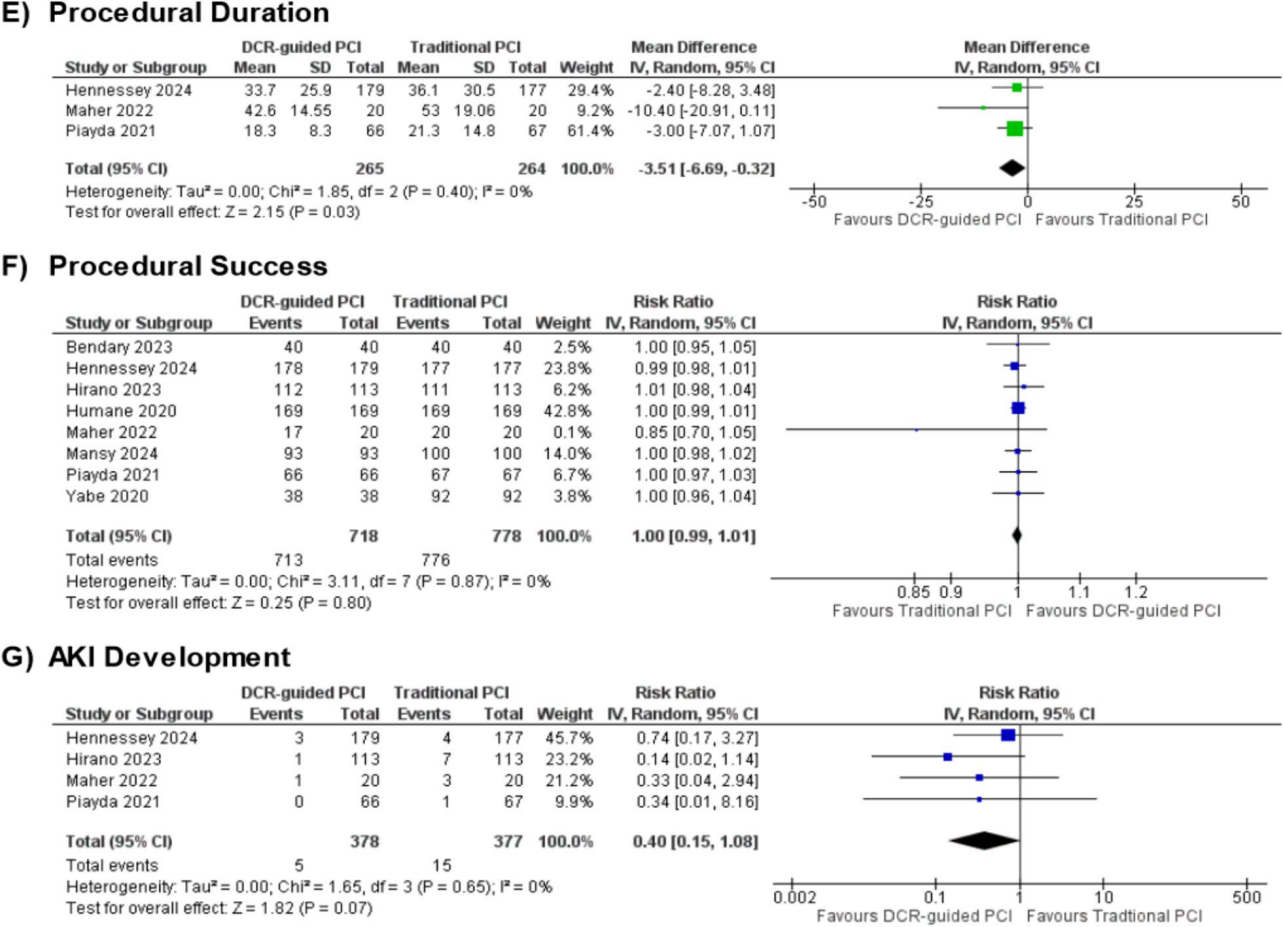
Meta-Analysis results continued

Three studies (n = 529) showed shorter procedure times with DCR-guided PCI (MD =  − 3.51 min, 95% CI [− 6.69, − 0.32], *p* = 0.03), with no observed heterogeneity (I^2^ = 0%, *p* = 0.87; Fig. 4E).

#### Procedural success

Across all eight studies (n = 1,496), procedural success did not differ significantly between groups (RR = 1.00, 95% CI [0.99, 1.01], *p* = 0.80), with no heterogeneity (I^2^ = 0%, *p* = 0.87; Fig. 4F).

#### Acute kidney injury (AKI)

Four studies (n = 755) reported AKI incidence. The pooled estimate showed a non-significant reduction in AKI with DCR-guided PCI (RR = 0.40, 95% CI [0.15, 1.08], *p* = 0.07), with no heterogeneity (I^2^ = 0%, *p* = 0.65; Fig. 4G).

## Discussion

In this meta-analysis, DCR-guided PCI was consistently associated with lower CMV, reduced radiation exposure (air kerma and dose–area product), shorter radiation duration, and shorter procedural times compared with conventional PCI. Importantly, these procedural benefits were achieved without compromising procedural success rates. No statistically significant difference in AKI incidence was observed, although a numerical reduction was noted in the DCR group.

CI-AKI is the third leading cause of in-hospital acute kidney injury, frequently encountered post-coronary angiography [18], and is significantly associated with adverse clinical outcomes and decreased survival rates [19, 20]. Preventive strategies for CI-AKI include appropriate risk stratification, using low-osmolar or iso-osmolar contrast media, minimizing CMV, and optimizing patients' hydration pre- and post-PCI. Renal-protective measures, such as N-acetylcysteine and sodium bicarbonate, are also crucial [18]. Of these strategies, reducing CMV remains the cornerstone procedural approach, given the well-established linear relationship between CMV and CI-AKI incidence [6]. DCR was introduced in 2017 by Philips Medical System as an innovative technique to enhance procedural guidance through dynamic 2D images without additional contrast [3]. Over the years, DCR has evolved with advances in imaging technologies, offering improved visualization, procedural efficiency, and safety, and potentially expanding to other interventional procedures, such as renal denervation [21]. These benefits are precious given the increasing risk profile of patients undergoing PCI [22].

Our analysis concluded that DCR significantly and consistently reduces CMV throughout the eight included studies. This reduction enhances PCI procedure safety by lowering the risk of CI-AKI. It enables completion of complex interventions without the constraints of CMV limits, which might otherwise require terminating or staging the procedure [23]. Although our analysis showed a lower incidence of AKI in the DCR-guided group, this difference did not reach statistical significance. This likely reflects limited statistical power, given the relatively small number of included patients and the restricted number of studies reporting AKI outcomes. In addition, two studies excluded patients with baseline CKD—the population at highest risk for CI-AKI and most likely to benefit from contrast reduction [18, 23, 24], resulting in an evidence base that may underrepresent those most susceptible to renal injury. Furthermore, the inclusion of a broad PCI population, rather than focusing specifically on high-risk groups such as patients with CKD or STEMI, may have diluted the ability to detect a clinically meaningful benefit [25]. Taken together, these factors suggest that the absence of statistical significance should not be interpreted as the absence of effect, but rather as reflecting current limitations in the available evidence.

Prioritizing radioprotection in cardiology departments is essential for cancer prevention, serves as a quality indicator, and protects the health of patients, doctors, and staff [26]. Fluoroscopy can deliver high radiation doses, particularly during complex interventional procedures like PCI, which often require prolonged exposure. Besides radiation risk, prolonged fluoroscopy is associated with increased in-hospital mortality, emergency surgery, and CI-AKI, as extended fluoroscopy time often reflects longer PCI duration [27]. To mitigate exposure, fluoroscopy should be performed at the lowest acceptable dose for the shortest necessary duration [28]. Our study concluded that DCR significantly reduced radiation exposure with a mean reduction in radiation duration averaging 9 min, as reported by Maher et al. [24], and almost 1 min in comparisons by Hennessey et al. [23] and Piyada et al.[29], while also significantly reducing Air kerma and DAP. This is particularly helpful with the increased frequency of complex PCIs in recent years, and increasing concerns over the radiation dose patients receive [30].

Procedural success was comparable between the two approaches, reaching 100% in both arms in five studies [2, 29, 31–33], and not significantly different in three [23, 24, 34], with a significantly shorter duration in the DCR-guided PCI overall, favoring DCR across all three included studies. This indicates that DCR improves procedural metrics without compromising PCI efficacy.

There was significant heterogeneity in the overall meta-analysis of CMV, air kerma, dose-area product, and radiation duration that could not be resolved through sensitivity analysis. Several patient- and procedure-related factors, such as the complexity of the affected lesions, influence the CMV used and the radiation delivered during the procedure [35–37]. Due to the novelty of this technology and the relatively low number of trials, our study included a wide patient population. It did not adjust for these factors between studies, potentially contributing to the observed heterogeneity among several studied outcomes.

In addition, several important procedural factors varied substantially across the included studies and were not consistently accounted for. The use of intravascular imaging (e.g., IVUS) ranged widely, and differences in lesion complexity, pre-dilation strategies, and procedural planning (ad hoc versus elective PCI) were also observed. These factors are known to independently influence contrast utilization, fluoroscopy time, and radiation exposure. As such, the observed reductions in contrast volume and radiation metrics may not be solely attributable to DCR itself, but may partly reflect differences in procedural complexity and operator practice patterns between studies. The inability to adjust for these variables limits the ability to fully isolate the independent effect of DCR. It likely contributes to the substantial heterogeneity observed across outcomes.

### Clinical implications

The procedural efficiencies demonstrated by DCR-guided PCI—namely, reduced contrast media use, shorter radiation exposure, and decreased procedural times—are not merely technical gains; they have direct patient safety implications. Even modest reductions in CMV may be clinically meaningful in high-risk groups such as patients with chronic kidney disease, diabetes, or advanced age. Prior studies suggest that exceeding contrast thresholds of 3.7 × estimated glomerular filtration rate (eGFR) markedly increases CI-AKI risk [6, 7], and that each additional 20–30 mL of contrast is associated with incremental increases in renal injury incidence [18]. The average CMV reduction observed in our analysis (~ 46 mL) is therefore likely to be clinically relevant, particularly in patients near their individual contrast limits.

Similarly, the observed reductions in air kerma and dose–area product may have long-term benefits for both patients and operators by lowering cumulative stochastic and deterministic radiation risks. In complex PCI cases, where procedural times and radiation exposure can escalate rapidly, integrating DCR could be particularly advantageous. These benefits align with the ALARA (As Low As Reasonably Achievable) principle for radiation safety, a recognized quality benchmark in catheterization laboratories [28].

From a systems perspective, DCR’s ability to enhance procedural efficiency without sacrificing success rates could translate into reduced cath-lab turnover times, lower cumulative occupational radiation exposure, and potential cost savings from avoided CI-AKI cases and shortened hospital stays. However, its adoption should initially be targeted toward patient populations and procedural contexts where the benefits are likely to be maximized—such as complex multivessel interventions, chronic total occlusions, and high-risk renal patients—until further evidence confirms its broad applicability.

### Strengths and limitations of the study

This meta-analysis represents the most comprehensive synthesis to date comparing DCR-guided PCI with conventional angiography-guided PCI. The protocol was prospectively registered, and the methodology strictly adhered to PRISMA and the Cochrane Handbook guidance, thereby strengthening the transparency and reproducibility of our findings. However, several limitations should be acknowledged.

First, significant heterogeneity persisted across several pooled outcomes—including CMV, air kerma, dose–area product, and radiation duration—that could not be resolved through sensitivity analysis. This heterogeneity likely reflects differences in patient risk profiles, lesion complexity, operator experience, and institutional protocols, which were not uniformly reported or adjusted for across studies.

Second, the evaluation of AKI—a key clinical endpoint given the central role of contrast reduction—was limited. Only a small number of studies reported AKI outcomes, and some explicitly excluded patients with CKD, who are at highest risk and most likely to benefit from contrast minimization strategies. Although a relative reduction in AKI was observed, it did not reach statistical significance, likely reflecting limited power rather than absence of effect. Consequently, the current evidence base is insufficient to determine whether reductions in contrast volume translate into meaningful renal protection, particularly in high-risk populations.

Third, most included studies were single-center investigations, potentially limiting the generalizability of results to broader healthcare settings.

Fourth, the included randomized trials demonstrated variable risk of bias, including potential industry sponsorship bias.

Fifth, a potential learning curve effect may exist, whereby operator familiarity with DCR influences procedural efficiency and outcomes over time; this factor was not consistently assessed across studies.

Finally, safety outcomes beyond AKI and procedural success were infrequently reported, limiting a comprehensive assessment of adverse events.

## Conclusion

Overall, DCR-guided PCI shows promise, particularly in settings where reducing contrast and radiation exposure is important. It appears to offer procedural advantages over conventional PCI, with comparable success rates and no indication of increased procedural risk. It was consistently associated with lower contrast use, reduced radiation exposure, and shorter procedural duration.

However, substantial heterogeneity across studies suggests that these findings reflect average effects across varied clinical settings, and the magnitude of benefit is likely to differ depending on patient characteristics and procedural factors. As such, the results should be interpreted with caution. In addition, the overall certainty of evidence is low, given the predominance of observational studies, the limited number of randomized trials, and their associated risk of bias, including potential industry influence. Evidence for clinical outcomes remains limited, as no significant reduction in AKI was observed, and major adverse cardiovascular events were not assessed.

Further large-scale, multicenter randomized studies are needed to understand better its impact across different patient populations and procedural contexts, and to clarify long-term clinical outcomes.

## Supplementary Information

Additional file1 (DOCX 14 KB)

## Data Availability

Data is provided within the manuscript or supplementary information files.

## Abbreviations

PCI: Percutaneous coronary intervention
CI: Confidence interval
CI-AKI: Contrast-induced acute kidney injury
CKD: Chronic kidney disease
CM: Contrast medium
CMV: Contrast media volume
IQR: Interquartile ranges
MD: Mean difference
PRISMA: Preferred reporting items for systematic reviews and meta-analyses
RCTs: Randomized controlled trials
RR: Risk ratio
SD: Standard deviation
STEMI: ST elevation myocardial infarction

## References

[1] Mungee (2024) MAPMAKRRS. Percutaneous coronary intervention. In: StatPearls editor. Treasure Island (FL): StatPearls Publishing

[2] Yabe T, Muramatsu T, Tsukahara R, Nakano M, Takimura H, Kawano M et al (2020) The impact of percutaneous coronary intervention using the novel dynamic coronary roadmap system. Heart Vessels 35:323–330. 10.1007/s00380-019-01502-1 31522247 10.1007/s00380-019-01502-1

[3] Almendarez M, Gurm HS, Mariani J, Montorfano M, Brilakis ES, Mehran R et al (2019) Procedural strategies to reduce the incidence of contrast-induced acute kidney injury during percutaneous coronary intervention. JACC Cardiovasc Interv 12:1877–1888. 10.1016/j.jcin.2019.04.055 31521648 10.1016/j.jcin.2019.04.055

[4] Chalikias G, Drosos I, Tziakas DN (2016) Contrast-induced acute kidney injury: an update. Cardiovasc Drugs Ther 30:215–228. 10.1007/s10557-015-6635-0 26780748 10.1007/s10557-015-6635-0

[5] Yang Y, George KC, Luo R, Cheng Y, Shang W, Ge S et al (2018) Contrast-induced acute kidney injury and adverse clinical outcomes risk in acute coronary syndrome patients undergoing percutaneous coronary intervention: a meta-analysis. BMC Nephrol 19:374. 10.1186/s12882-018-1161-5 30577763 10.1186/s12882-018-1161-5 PMC6303898

[6] Tehrani S, Laing C, Yellon DM, Hausenloy DJ (2013) Contrast-induced acute kidney injury following PCI. Eur J Clin Invest 43:483–490. 10.1111/eci.12061 23441924 10.1111/eci.12061

[7] Azzalini L, Vilca LM, Lombardo F, Poletti E, Laricchia A, Beneduce A et al (2018) Incidence of contrast-induced acute kidney injury in a large cohort of all-comers undergoing percutaneous coronary intervention: comparison of five contrast media. Int J Cardiol 273:69–73. 10.1016/j.ijcard.2018.08.097 30196995 10.1016/j.ijcard.2018.08.097

[8] Piayda K, Kleinebrecht L, Afzal S, Bullens R, ter Horst I, Polzin A et al (2018) Dynamic coronary roadmapping during percutaneous coronary intervention: a feasibility study. Eur J Med Res 23:36. 10.1186/s40001-018-0333-x 30064500 10.1186/s40001-018-0333-x PMC6069549

[9] Hao R, Zhang Q, Xu Z, Tang L, Yang Z, Cao K et al (2013) Magnetic navigation system and CT roadmap-assisted percutaneous coronary intervention: a comparison to the conventional approach. J Invasive Cardiol 25:177–181 23549490

[10] Ma H, Smal I, Daemen J, van Walsum T (2020) Dynamic coronary roadmapping via catheter tip tracking in X-ray fluoroscopy with deep learning based Bayesian filtering. Med Image Anal 61:101634. 10.1016/j.media.2020.101634 31978856 10.1016/j.media.2020.101634

[11] Page MJ, McKenzie JE, Bossuyt PM, Boutron I, Hoffmann TC, Mulrow CD, The PRISMA et al (2020) statement: an updated guideline for reporting systematic reviews. BMJ 2021:n71. 10.1136/bmj.n71 10.1136/bmj.n71 PMC8005924 33782057

[12] Higgins JPT, Thomas J, Chandler J, Cumpston M, Li T, Page MJ WV, (Ed.) (2023) Cochrane handbook for systematic reviews of interventions. 6.4. Cochrane, 2023

[13] Higgins JPT, Altman DG, Gotzsche PC, Juni P, Moher D, Oxman AD et al (2011) The cochrane collaboration’s tool for assessing risk of bias in randomised trials. BMJ 343:d5928–d5928. 10.1136/bmj.d5928 22008217 10.1136/bmj.d5928 PMC3196245

[14] Stang A (2010) Critical evaluation of the Newcastle-Ottawa scale for the assessment of the quality of nonrandomized studies in meta-analyses. Eur J Epidemiol 25:603–605. 10.1007/s10654-010-9491-z 20652370 10.1007/s10654-010-9491-z

[15] The Cochrane Collaboration (2020) RevMan. The Cochrane Collaboration

[16] Wan X, Wang W, Liu J, Tong T (2014) Estimating the sample mean and standard deviation from the sample size, median, range and/or interquartile range. BMC Med Res Methodol 14:135. 10.1186/1471-2288-14-135 25524443 10.1186/1471-2288-14-135 PMC4383202

[17] Hozo SP, Djulbegovic B, Hozo I (2005) Estimating the mean and variance from the median, range, and the size of a sample. BMC Med Res Methodol 5:13. 10.1186/1471-2288-5-13 15840177 10.1186/1471-2288-5-13 PMC1097734

[18] Shabbir A et al (2015) Contrast-induced nephropathy in PCI: an evidence-based approach to prevention. Br J Cardiol. 10.5837/bjc.2015.001

[19] Zhang Y, Liu D, Zhou Y, Lou J (2021) Acute kidney injury in patients with acute coronary syndrome after percutaneous coronary intervention: pathophysiologies, risk factors, and preventive measures. Cardiology 146:678–689. 10.1159/000517991 34348269 10.1159/000517991

[20] Gupta R, Bang T (2010) Prevention of contrast-induced nephropathy (CIN) in interventional radiology practice. Semin Intervent Radiol 27:348–359. 10.1055/s-0030-1267860 22550376 10.1055/s-0030-1267860 PMC3324211

[21] Gonzálvez-García A, Jurado-Román A, Tébar-Márquez D, Jiménez-Valero S, Galeote G, Rivero-Santana B et al (2024) Role of dynamic road-mapping in renal denervation procedures. J Invasive Cardiol. 10.25270/jic/24.00064 38517884 10.25270/jic/24.00064

[22] Kirtane AJ, Doshi D, Leon MB, Lasala JM, Ohman EM, O’Neill WW et al (2016) Treatment of higher-risk patients with an indication for revascularization: evolution within the field of contemporary percutaneous coronary intervention. Circulation 134:422–431. 10.1161/CIRCULATIONAHA.116.022061 27482004 10.1161/CIRCULATIONAHA.116.022061 PMC9117111

[23] Hennessey B, Danenberg H, De Vroey F, Kirtane AJ, Parikh M, Karmpaliotis D et al (2024) Dynamic coronary roadmap versus standard angiography for percutaneous coronary intervention: the randomised, multicentre DCR4Contrast trial. EuroIntervention 20:e198–e206. 10.4244/EIJ-D-23-00460 38343370 10.4244/EIJ-D-23-00460 PMC10851082

[24] Maher M, Zarif B, Elgamal A, Khairy H, Magdy A (2022) Dynamic coronary roadmap for contrast, and radiation time reduction during coronary intervention (DRM-COR). Am J Health Med Nurs Pract 7:32–39. 10.47672/ajhmn.1298

[25] Tsai TT, Patel UD, Chang TI, Kennedy KF, Masoudi FA, Matheny ME et al (2014) Contemporary incidence, predictors, and outcomes of acute kidney injury in patients undergoing percutaneous coronary interventions. JACC Cardiovasc Interv 7:1–9. 10.1016/j.jcin.2013.06.016 24456715 10.1016/j.jcin.2013.06.016 PMC4122507

[26] Picano E, Piccaluga E, Padovani R, Antonio Traino C, Grazia Andreassi M, Guagliumi G (2014) Risks related to fluoroscopy radiation associated with electrophysiology procedures. J Atr Fibrillation 7:1044. 10.4022/jafib.1044 27957094 10.4022/jafib.1044 PMC5135251

[27] Tajti P, Ayoub M, Nuehrenberg T, Ferenc M, Behnes M, Buettner HJ et al (2021) Association of prolonged fluoroscopy time with procedural success of percutaneous coronary intervention for stable coronary artery disease with and without chronic total occlusion. J Clin Med 10:1486. 10.3390/jcm10071486 33916666 10.3390/jcm10071486 PMC8038393

[28] U.S. Food and Drug Administration (2023) Fluoroscopy. [cited 14 July 2024]. Available: https://www.fda.gov/radiation-emitting-products/medical-x-ray-imaging/fluoroscopy#benefitsrisks

[29] Piayda K, Phinicarides R, Afzal S, Veulemans V, Jung C, Bönner F et al (2021) Dynamic coronary roadmap in percutaneous coronary intervention. JACC Cardiovasc Interv 14:2523–2525. 10.1016/j.jcin.2021.08.068 34743899 10.1016/j.jcin.2021.08.068

[30] Georges J-L, Karam N, Tafflet M, Livarek B, Bataille S, Loyeau A et al (2017) Time-course reduction in patient exposure to radiation from coronary interventional procedures. Circ Cardiovasc Interv. 10.1161/CIRCINTERVENTIONS.117.005268 28801540 10.1161/CIRCINTERVENTIONS.117.005268

[31] Bendary A, Mahmoud D, Attia A, Elrabbat K (2023) Value of the new dynamic coronary roadmap system in percutaneous coronary intervention for patients with chronic coronary syndrome. Iran Heart J 24:34–41

[32] Mansy A, Allam S, Hussien M, Solyman O (2024) Value of the novel dynamic coronary roadmap in percutaneous coronary intervention. Cardiol Cardiovasc Res. 10.11648/ccr.20240801.12

[33] Humane DR, Rakesh T, Sandip R, Kavita R, Nandita R, Darshana D et al (2020) International journal of current medical and comparative study of pci using dynamic coronary roadmap vs PCI without using dynamic coronary roadmap on radiation dose. Contrast Vol Fluoroscopy Time 6:5358–5362

[34] Hirano S, Yabe T, Oka Y, Kojima Y, Aikawa H, Noike R et al (2023) Clinical outcomes of patients with chronic kidney disease undergoing percutaneous coronary interventions with a novel dynamic coronary roadmap system. Int Heart J 64:23–213. 10.1536/ihj.23-213 10.1536/ihj.23-213 37704405

[35] Stocker TS, Abdel-Wahab M, Möllmann H, Deseive S, Massberg S, Hausleiter J (2022) Trends and predictors of radiation exposure in percutaneous coronary intervention: the protection VIII study. EuroIntervention 18:e324–e332. 10.4244/EIJ-D-21-00856 35076020 10.4244/EIJ-D-21-00856 PMC9912963

[36] Christakopoulos GE, Karmpaliotis D, Alaswad K, Yeh RW, Jaffer FA, Wyman RM et al (2016) Contrast utilization during chronic total occlusion percutaneous coronary intervention: insights from a contemporary multicenter registry. J Invasive Cardiol 28:288–294 27342206 PMC5705198

[37] Fetterly KA, Lennon RJ, Bell MR, Holmes DR, Rihal CS (2011) Clinical determinants of radiation dose in percutaneous coronary interventional procedures. JACC Cardiovasc Interv 4:336–343. 10.1016/j.jcin.2010.10.014 21435613 10.1016/j.jcin.2010.10.014

