# Supplementary material for: Dynamic coronary roadmap-guided versus traditional percutaneous coronary intervention techniques in contrast medium volume reduction: a systematic review and meta-analysis
Source: Egypt Heart J. 2026 Jul 1;78:51. doi: 10.1186/s43044-026-00763-2 (PMC13319315; doi:10.1186/s43044-026-00763-2)
Supplement: Supplementary file 1 — Additional file1 (DOCX 14 KB) [file 43044_2026_763_MOESM1_ESM.docx]

| Cochrane Library | ((Roadmap Fusion Imaging) OR (Dynamic Coronary Roadmap) OR (DCR)) AND ((angiography) OR (Arteriography) OR (Angiogram) OR (Percutaneous coronary intervention) OR (percutaneous coronary revascularisation) OR (PCI) OR (Coronary Angiography)) |
| --- | --- |
| Embase  Pubmed | ('roadmap fusion imaging' OR (roadmap AND ('fusion'/exp OR fusion) AND ('imaging'/exp OR imaging)) OR 'dynamic coronary roadmap' OR (('dynamic'/exp OR dynamic) AND coronary AND roadmap) OR dcr) AND ('angiography'/exp OR angiography OR 'arteriography'/exp OR arteriography OR 'angiogram'/exp OR angiogram OR 'percutaneous coronary intervention'/exp OR 'percutaneous coronary intervention' OR (percutaneous AND coronary AND ('intervention'/exp OR intervention)) OR 'percutaneous coronary revascularisation' OR (percutaneous AND coronary AND ('revascularisation'/exp OR revascularisation)) OR pci OR 'coronary angiography'/exp OR 'coronary angiography' OR (coronary AND ('angiography'/exp OR angiography))) |
| Scopus  Web of Science | TITLE-ABS-KEY ((( roadmap AND fusion AND imaging ) OR ( dynamic AND coronary AND roadmap ) OR ( dcr ) ) AND ( ( angiography ) OR ( arteriography ) OR ( angiogram ) OR ( percutaneous AND coronary AND intervention ) OR ( percutaneous AND coronary AND revascularisation ) OR ( pci ) OR ( coronary AND angiography ) ) ) |

Table S1. Used Search strategy in databases.
